# Supplementary material for: Expression of Transcription Factor ZBTB20 in the Adult Primate Neurogenic Niche under Physiological Conditions or after Ischemia
Source: Genes (Basel). 2022 Aug 29;13(9):1559. doi: 10.3390/genes13091559 (PMC9498320; doi:10.3390/genes13091559)
Supplement: Supplementary file 1 [file genes-13-01559-s001.zip › genes-1823012-supplementary.pdf]

Stoyanov D et al., Expression of Transcription Factor ZBTB20 in the Adult Primate Neurogenic Niche under Physiological Conditions or after Ischemia Supplementary Figures S1 and S2.

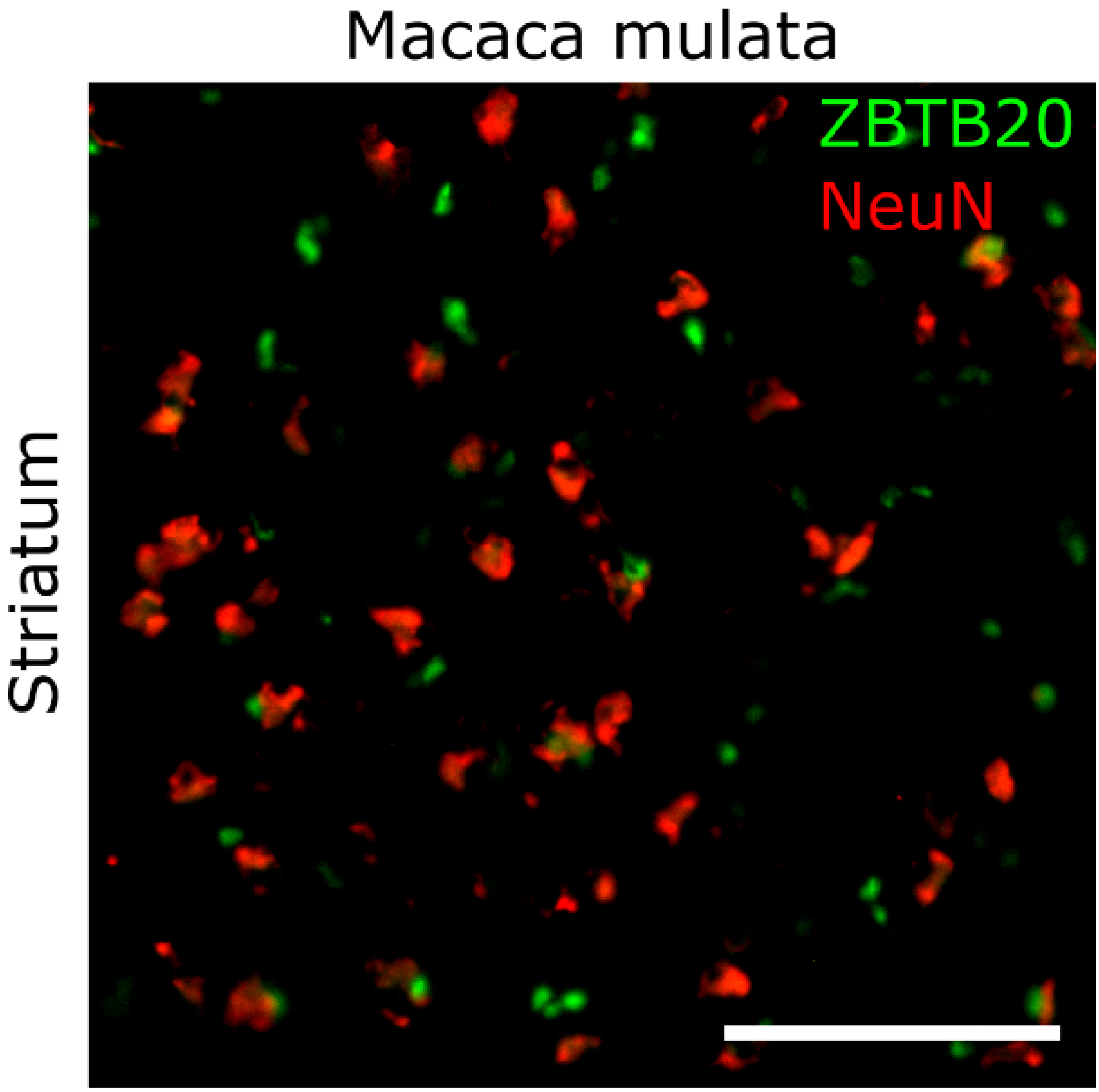

Figure S1. ZBTB20 is absent in mature striatal neurons Double-staining for ZBTB20 and NeuN in macaque striatum shows absence of double-positive cells. Scale bar: 100μm.

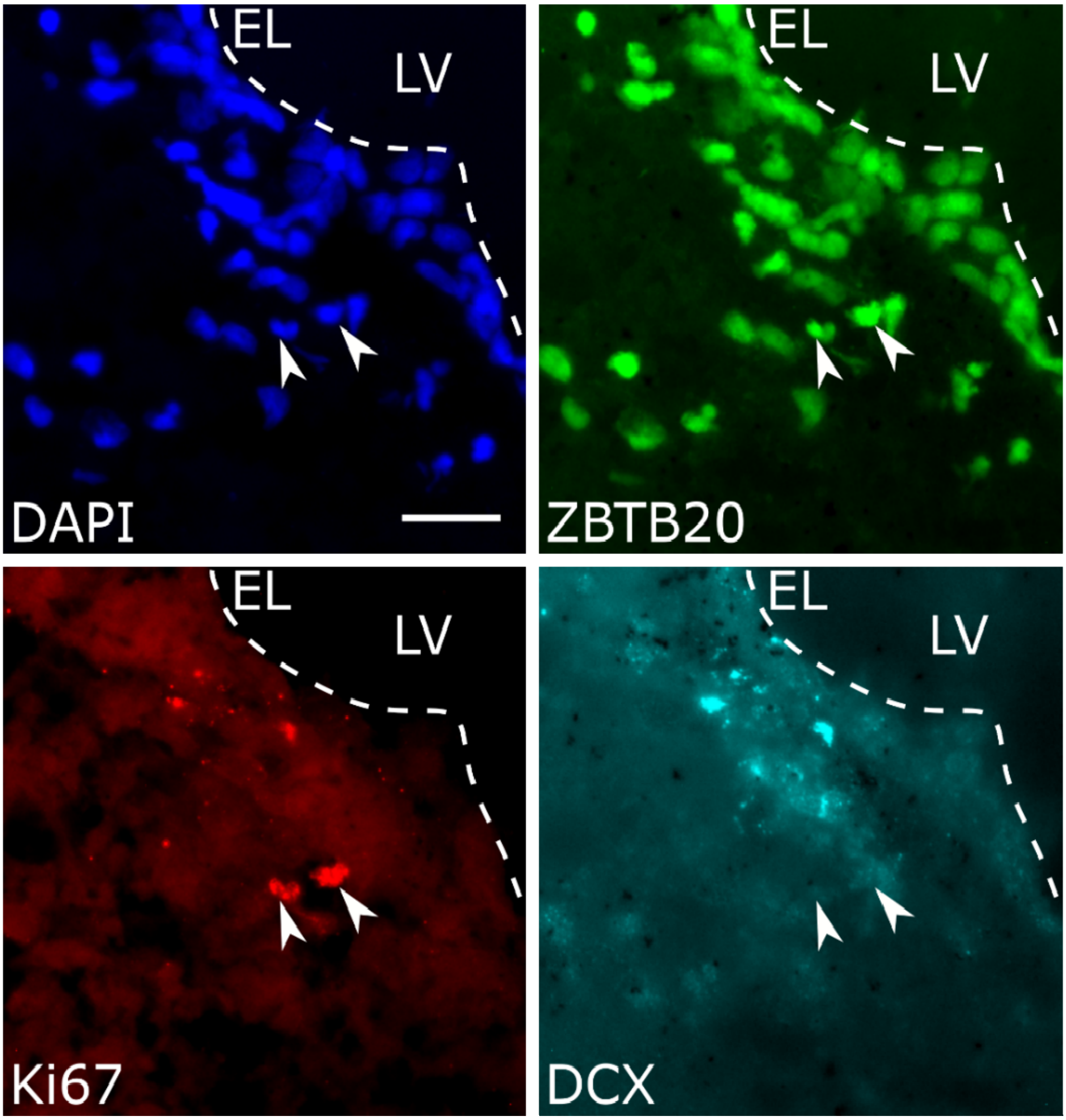

Figure S2. Combinatorial labeling of ZBTB20 with Ki67 and DCX. Triple-staining for Ki67, ZBTB20 and DCX in macaque monkey SEL shows presence of $Ki67^{+}/ZBTB20^{+}$ cells which are negative for DCX (arrowheads). EL – ependymal layer, LV – lateral ventricle. Scale bar: 20μm.
